# Amyloid fibrils of the Als5p-derived peptide NH_2_-SNGIVIVATTRTV-COOH influence the biofilm formation of *Candida albicans* by shape-edging microcolony morphology

**DOI:** 10.1080/21505594.2025.2597576

**Published:** 2025-12-10

**Authors:** Daniel Gruber, Jan-Christoph Walter, Grigory Bolotnikov, Benedikt Miegel, Elisabeth Bierla, Andreas Bellmann, Christina Sandra Einsiedler, Amelie Therese Eiblmayr, Mauritz Maser, Denise Obert, Elisa Lin Men Qi Sun, Jonas Schnaubelt, Seda Söylemezgiller, Bianca Andrea Widy, Barbara Spellerberg, Steffen Stenger, Armando Rodríguez-Alfonso, Nico Preising, Ludger Ständker, Carolina Firacative, Ann-Kathrin Kissmann, Frank Rosenau

**Affiliations:** a Institute of Pharmaceutical Biotechnology, Ulm University, Ulm, Germany; b Department Biology, Technische Universität München, München, Germany; c Institute of Medical Microbiology and Hygiene, University Clinic of Ulm, Ulm, Germany; d Core Facility for Functional Peptidomics (CFP), Faculty of Medicine, Ulm University, Ulm, Germany; e Studies in Translational Microbiology and Emerging Diseases (MICROS) Research Group, School of Medicine and Health Sciences, Universidad de Rosario, Bogota, Colombia; f Faculty of Medicine and Dentistry, Danube Private University, Krems an der Donau, Austria

**Keywords:** Amyloid fibrils, Als5p fibril-forming peptide, *candida albicans*, image analysis, thioflavin T assay

## Abstract

*Candida* species are major contributors to nosocomial infections, with biofilm formation being a critical virulence factor that enables persistence in clinical settings and resistance to antifungal therapies. Central to biofilm development is the adhesion of fungal cells, a process mediated by surface proteins such as Als5p in *Candida albicans*. The amyloid-forming peptide sequence within Als5p (^322^SNGIVIVATTRTV^334^) has been implicated in mediating adhesion and biofilm formation; however, its role in shaping the biofilm architecture has not been fully elucidated. In this study, we demonstrated that the addition of Als5pFP promoted biomass accumulation in *C. albicans* biofilms under laboratory conditions, including complex media and at temperatures compatible with clinical biofilm assays. Using advanced image analysis of microscopy images, we show that the Als5p peptide induces a distinct morphological effect on biofilms: a shape-edging of microcolony structures, characterized by the concentration of fungal cells into denser aggregates and the reduction of cells in intermediate spaces. These observations suggest a potential role of amyloid-like fibrils formed by the Als5p peptide in influencing the spatial organization of *C. albicans* biofilms. This discovery presents a novel aspect on how these fibrils affect the biofilm architecture extending beyond previous studies, which primarily focused on biomass accumulation. Our findings contribute to the understanding of the architectural development of *C. albicans* biofilms and provide a foundation for future research aimed at targeting the amyloid structures within fungal biofilms. Furthermore, the results may support the design of biofilm-targeting antifungal agents and development of biosensors for monitoring amyloid formation during infection.

## Introduction

**Figure 1.**
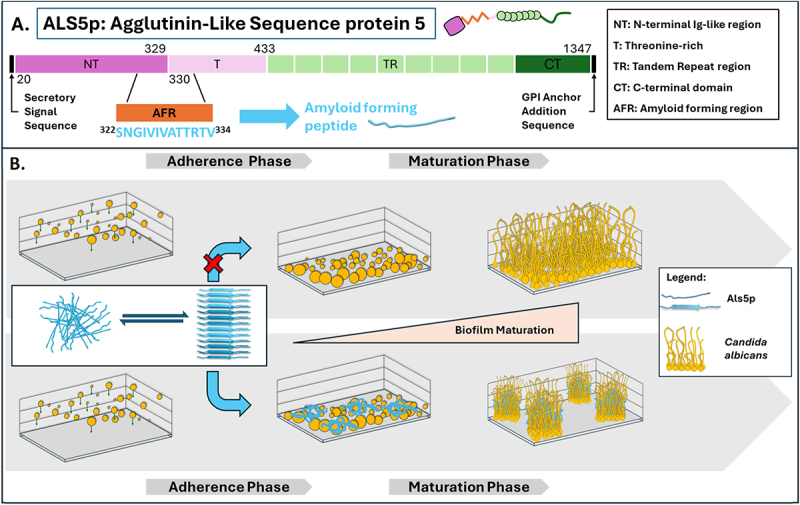
Structural and functional overview of Als5p and its role in amyloid-mediated adhesion and biofilm formation. (A) schematic representation of Als5p (agglutinin-like sequence protein 5) domains, including the N-terminal Ig-like region (nt), threonine-rich (t), tandem repeat (tr) regions, and the C-terminal (ct) domain. The amyloid-forming region (afr), highlighted in orange, contains a conserved amyloid-forming peptide (residues 322–334) shown in blue (^322^SNGIVIVATTRTV^334^) [59]. (B) proposed model for Als5pFP-mediated adherence and biofilm maturation, followed by amyloid fibril formation that stabilizes mature biofilms. The addition of Als5pFP facilitates a shape-edging of microcolony morphology [60].

*Candida* species, which are considered benign commensals within the human microbiota, are opportunistic pathogens in certain clinical conditions. They typically colonize the mucosal surfaces of the gastrointestinal tract and the skin without causing harm [1,2]. [1,2]Disruptions such as immunosuppression, surgical interventions, antibiotic use, or long intensive care unit stays, can trigger a shift toward pathogenicity [3–12]. This transition is particularly dangerous in the form of invasive candidiasis, which is a severe condition associated with mortality rates as high as 55% [13–15]. Among fungal pathogens, *Candida* spp. are a significant contributor to the global health burden. Despite being often neglected in infectious diseases, fungi are implicated in approximately 1.5 million deaths annually, outpacing tuberculosis in some contexts [16]. The World Health Organization (WHO) has highlighted this issue by placing *Candida* species on the list of the most concerning fungal threats owing to their resistance and clinical relevance [17]. Although nearly 200 *Candida* species and related genera have been described, only approximately 15 have been linked to human disease [5,18]. A subset of six species, three of which were recently reclassified, dominate the clinical scene: *Candida albicans, Candidozyma auris* (formerly *Candida auris*), *Candida parapsilosis, Candida tropicalis, Nakaseomyces glabratus* (formerly *C. glabrata*), *Pichia kudriavzevii* (formerly *Candida krusei*) [19–24]. The distribution and prevalence of these diseases are influenced by patient demographics and environmental factors. For instance, *C. parapsilosis* is prevalent in neonates and is often isolated from catheter-related infections [25]. Bloodstream infections caused by *Candida* are among the most frequent nosocomial infections in hospitals, particularly among immunocompromised and critically ill patients [26–28]. In addition to clinical settings, *Candida* causes widespread mucosal infections, with vulvovaginal candidiasis being one of the most common, affecting the majority of women at least once in their lives, with many experiencing recurrent episodes [18]. The COVID-19 pandemic has further complicated these infection patterns, potentially expanding the population’s vulnerability to *Candida* infections [29]. *C. albicans* remains the most studied and virulent species within the genus, largely because of its exceptional ability to adapt to and invade host tissues [26]. One of its defining traits is morphological plasticity, which means that it can alternate between yeast and filamentous forms, enhancing tissue penetration and immune evasion [30–32]. Its genomic adaptability supports rapid phenotypic shifts under environmental stress [33–35]. Additionally, the secretion of hydrolytic enzymes [36,37] and formation of robust biofilms contribute significantly to pathogenic arsenal [38,39]. Biofilm formation is a common and highly organized microbial lifestyle that offers protection from environmental and host-derived stressors. It is particularly relevant in pathogenic fungi, such as *Candida* species, where it contributes significantly to persistence and resistance in clinical settings [40–44]. Biofilms are formed through a multistep developmental process, including adhesion, early growth, maturation, and eventual dispersal of cells [45]. The initial adhesion phase, occurring within the first 10–12 h, involves planktonic cells attached to a surface and aggregating into microcolonies facilitated by surface adhesins [46,47]. *C. albicans* typically adheres to mucosal epithelial cells [48], whereas others, such as *C. parapsilosis*, are more often associated with abiotic materials such as central venous catheters [49,50]. As the biofilm matures, morphological changes occur (e.g. hyphal formation in *C. albicans*) and extracellular polymeric substances (EPSs) are produced, which form a matrix critical for structure, nutrient retention, and drug resistance [46,51]. Biofilm development is influenced by various host and environmental factors including nutrient availability, fluid shear, oxygen levels, and host immune status [46,52–55]. Moreover, specific metabolic and regulatory genes, such as those encoding alcohol dehydrogenases, modulate the biofilm architecture and virulence [56,57]. *C. albicans* expresses cell wall proteins, which play essential roles in mediating adhesion, as the initial step of biofilm formation, and the, most prominently established, the adhesin Als5p. Als5p belongs to a family of proteins identified in a variety of pathogenic yeasts [58]. Bioinformatic analysis revealed the organization of Als proteins in four conserved domains (NT, T, TR and CT) (Figure 1(A)) with the NT and T domains harboring the so-called amyloid forming region (AFR). They are suspected to mediate amyloid fibril forming capability and, in turn, adhesive properties of the cell to cause effects on biofilm formation (Figure 1(B)) [61–63]. According to the current model, binding of protein ligands either from the host or yeast cell to the additional PBC domain (peptide-binding cavity) located in the NT domain results in conformational changes that expose the AFR region and prepare the Als protein for fibril formation [64]. Convincing experimental evidence came from the study of Lipke *et al*., which demonstrated that upon heterologous expression of the Als5 gene in *Saccharomyces cerevisiae* cell aggregation occurred, which could be completely inhibited by a single point mutation (i.e. exchange of Valine to Asparagine at position 326 of the complete protein resembling position 5 within the peptide sequence (^322^SNGIVIVATTRTV^334^) of Als5p) [65]. To the best of our knowledge, the resulting synthetic peptide has no distinct name except for its full sequence; therefore, we suggest the name Als5pFP (Fibril forming Peptide). Interestingly, a modified version of the Als5pFP synthetic peptide was also functional as a fibril formation perturbant [60]. The fibril formation properties of Als5pFP were clearly demonstrated experimentally *in vitro* in the study by PN Lipke and colleagues [66] and its influence on biofilm formation by *C. albicans* resulting in increased deposition of biomass [65]. Here, we demonstrate that fibril formation of this type is not only possible in pure water, but can also be observed in complex microbiological media and at temperatures up to 37°C, as required for biofilm formation assays according to clinical standards [67] that were already applied in several other studies of our group for the development and characterization of diverse antimicrobial peptides [68–72]. In this experimental setting, the addition of Als5pFP also resulted in increased deposition of *C. albicans* biomass; however, analyses of microscopic photographs via a new computational image analysis workflow revealed significant alterations in biofilm morphology on surfaces. In our opinion, these alterations are best described as shape-edging or “sharpening” of microcolonies by the concentration of single cells from intermediate spaces into the microcolonies. While previous studies primarily linked Als5pFP to biomass accumulation our findings extend this understanding by demonstrating its distinct role in microcolony shape-edging and morphological reorganization, which extends current knowledge of Als5p-fibrils by the addition of its effect on *C. albicans* biofilm architecture.

## Materials and methods

### Materials

Acetic acid, crystal violet, 3-(N-morpholino) propanesulfonic acid (MOPS), and paraformaldehyde were obtained from Carl Roth GmbH (Karlsruhe, Germany). RPMI-1640 medium containing l-glutamine was acquired from Thermo Fisher Scientific (Waltham, MA, USA). Thioflavin T was obtained from Sigma-Aldrich (St. Louis, MO, USA). Phosphate-buffered saline (PBS) was acquired from Life Technologies (Carlsbad, CA).

### Peptide synthesis

Als5pFP was produced according to the approach reported by Mildenberger *et al*. (2024) [73]. In summary, the peptide was synthesized using automated solid-phase peptide synthesis, purified by high-performance liquid chromatography (HPLC), and its quality was confirmed by liquid chromatography-mass spectrometry (LC-MS) and matrix-assisted laser desorption/ionization time-of-flight (MALDI-TOF) mass spectrometry. The Als5pFP peptide was produced through chemical synthesis by the Core Facility Functional Peptidomics at the University of Ulm (Ulm, Germany).

### Transmission electron microscopy (TEM)

The specimens were prepared as previously described [74]. For this, 5 μL of the respective sample suspension was left to adhere onto formvar and carbon-coated 200 mesh copper for 1 min at room temperature. The grid was then washed three times with aqua bidest, stained with 2% (*w/v*) uranyl acetate solution in aqua bidest, and dried. Finally, the dried grid was examined under a JEM-1400 transmission electron microscope (JEOL, Tokyo, Japan) at 120 kV.

### Cultivation of *C. albicans*

The laboratory strain *C. albicans* (ATCC 90,028) was purchased from the IPK Laboratory of Medical Mycology. For suspension cultures, 5 mL of RPMI-1640 supplemented with l-glutamine was inoculated from cryocultures and grown at 37°C with orbital shaking at 150 rpm for 16 h.

### Biofilm assay/crystal-violet-assay with microscopic imaging

Biofilm formation and quantification were performed in triplicate to test the effect of Als5pFP peptide on *C. albicans* cells. 2.5 × 10^3^ *C. albicans* yeast cells (2.5 × 103) were seeded in 200 μL RPMI-1640 medium supplemented with L-glutamine (Sigma-Aldrich) into flat-bottomed 96-well polystyrene microtiter plates (Sarstedt AG & Co., KG, Nümbrecht, Germany). Als5pFP was added at a final concentration of 0.05 μg/mL and 0.5 μg/mL directly into the wells containing *C. albicans* cells at the time of seeding. Wells without Als5pFP served as the controls. The plates were incubated for 24 h at 37°C without agitation to allow biofilm formation. After 24 h, planktonic (non-adherent) cells were carefully removed, and the wells were washed twice with 200 μL sterile water to eliminate loosely attached cells. Biofilm mass was quantified using the crystal violet staining method described by George O´Toole [75]. The biofilms were stained with 200 μL of 0.1% (*w/v*) crystal violet solution for 15 min at room temperature. Excess stain was removed and the wells were washed twice with 200 μL of sterile water. The plates were air-dried for 24 h at room temperature. The stained biofilms were solubilized by adding 200 μL of 30% (*v/v*) acetic acid to each well and incubating for 15 min at 25°C. The resulting solution was transferred to a new 96-well plate and the absorbance was measured at 560 nm using a Tecan Infinite F200 plate reader (Tecan Group Ltd., Switzerland). Additionally, biofilm morphology was analyzed using light microscopy. Images of biofilms were acquired using a Leica DMi8 Fluorescence Microscope at 20x magnification.

### Quantitative image analysis of biofilms using Fiji (ImageJ)

Microscopic images of *C. albicans* biofilms were analyzed using ImageJ/Fiji (version 1.54f; https://imagej.net/software/fiji/downloads). to assess the structural differences following treatment with Als5pFP. After 24 hours of biofilm formation, the analyzed images were taken under three conditions: no Als5pFP (control), 0.05 µg/mL Als5pFP and 0.5 µg/mL Als5pFP. For each image, grayscale intensity values were extracted using the histogram function, which generates a distribution of pixel counts across gray value bins, from low numbers being darker to higher numbers being brighter. The mean gray value of each image was recorded to estimate the average image brightness. To analyze the background signal more precisely, each histogram was examined to identify the background peak, which is defined as the most prominent high-intensity region of the histogram corresponding to image areas without visible *C. albicans* cells (bright background). Only pixel values within the full width at half maximum of the background peak in the histogram were selected for further analysis. Additionally, the mean gray value of the background peak was recorded to verify that the background gray values were consistent between the images. A threshold function was applied to Fiji to visualize and quantify the background distribution across each image. All pixels falling within the defined background gray value range (half maximum of the background peak) were selected and highlighted in red, excluding darker regions containing cells or brighter, potentially overexposed areas. This enabled a clear demarcation of the background-only regions. The percentage of the total image area corresponding to the thresholded background was calculated, allowing for a quantitative comparison of the background coverage across conditions, reflecting the extent and spatial distribution of biofilm growth.

### Thioflavin T (ThT) assay for monitoring Als5pFP fibril formation

Fibril formation by Als5pFP was assessed over 24 h using a Thioflavin T (ThT) fluorescence assay. ThT was prepared as a 0.8 mg/mL stock solution in phosphate-buffered saline (PBS), filtered through a 0.2 µm syringe filter, and stored protected from light at 4°C. Als5pFP was dissolved at a final concentration of 0.5 mg/mL in precooled (4°C) sterile water and RPMI medium. Two experimental conditions were tested: (1) continuous agitation at 50 rpm at 4°C (H_2_O and RPMI), and (2) incubation without agitation at 4°C (H_2_ O and RPMI). Aliquots were drawn directly from the Als5pFP solution. Samples were collected at five time points: immediately after complete dissolution of Als5pFP (~5 min vortex) and after 1, 3, 6, and 24 h. After each sampling, fluorescence measurements were performed immediately to assess fibril formation at the time of sampling. For fluorescence measurements, samples were transferred into black 384-well microplates (Sarstedt AG & Co., KG, Nümbrecht, Germany). ThT was added to each well at a final concentration of 20 µM (1 µL ThT solution per well), mixed gently, and incubated at room temperature for 15 min. Fluorescence was measured using a Tecan SPARK microplate reader (Tecan Group Ltd., Männedorf, Switzerland) with excitation at 450 nm and emission at 490 nm (bandwidth, 10 nm; gain, 70). Data was analyzed using GraphPad Prism (GraphPad Prism version 9.0 for Windows, GraphPad Software, www.graphpad.com). In addition to the 4°C conditions, further experiments were conducted to mimic the physiological environment of biofilm assays. Als5pFP was dissolved in RPMI medium at a final concentration of 0.5 mg/mL and incubated without agitation at 37°C. ThT fluorescence was measured using a previously described protocol. Two experimental variants were tested under the following conditions: (1) pure RPMI containing only Als5pFP and (2) RPMI containing Als5pFP together with *Candida* albicans cells, to assess the effect of cells on fibril formation. In both cases, measurements were performed immediately after dissolving Als5pFP in H_2_O or RPMI with cells, and again after 24 h of incubation. The rationale for exploring different incubation conditions was based on a previous study by PN Lipke and colleagues (2008) [66], who generated amyloid from Als peptides by stirring samples for 2 days at 5 °C, followed by static incubation for another 2 days at the same temperature. Their protocol was adopted from a previous study on tau peptide gelation [76].

### Unbiased volunteer based image analysis of biofilm microscopy images

To support an unbiased analysis of biofilm morphology under different Als5pFP concentrations, a volunteers unbiased Image Analysis approach was performed. Ten independent volunteers (i.e. student coworkers, who had agreed to invest experimental time) with no prior knowledge of the experimental hypothesis or expected outcomes were recruited for image analysis. All ten volunteers had academic or professional backgrounds in life sciences, such as medicine, biology, biochemistry, pharmaceutical biotechnology, or psychology, ensuring a basic familiarity with biological imaging without prior exposure to the specific experimental context. In this sense the volunteers were not participants in a medical study, but have been listed as authors based on their experimental and editorial contribution. Volunteers were provided with microscopic images of *C. albicans* biofilms treated with 0 mg/mL (control), 0.05 mg/mL and 0.5 µg/mL Als5pFP. Prior to annotation, all participants were instructed to examine all images from each condition to familiarize themselves with the range of gray values and structural patterns present across the dataset. After this familiarization phase, each volunteer was tasked with identifying and manually circling the darkest visible cluster(s) in each image using ImageJ/Fiji (version 1.54f) with a self-written macro (available upon request), measuring the area of the circled clustered for each image. Volunteers were instructed to mark only regions if they consistently represented dark areas compared with the other images. These manually annotated regions were termed “Volunteers Circled Areas” (VCAs). Following the completion of all annotations, VCA data were compiled and analyzed. For each image, the individual areas of all the annotated circles were summed to obtain the total VCA per image. The cumulative VCA was then divided by the total image area to calculate the relative VCA coverage, expressed as a percentage. This allowed for a standardized comparison of the proportion of dark regions across different Als5pFP treatment conditions. In addition to the area measurements, the average gray value within each manually annotated VCAs was determined. For each image, the gray values of all selected regions were extracted and averaged across all volunteers. This allowed for a direct comparison of the intensities of the selected clusters across different Als5pFP concentrations and among different volunteers. The resulting gray value data provided insights into the consistency of region selection and perceived contrast of biofilm structures under each treatment condition.

### Statistical analysis

Statistical analysis was performed using the unpaired Student’s t-test in the GraphPad Prism software. Differences were considered statistically significant at *p*-values less than 0.05. Statistical significance is indicated as follows: n.s., not significant; *p < 0.05, **p < 0.01, ***p < 0.001, and ****p < 0.0001.

## Results

**Figure 2.**
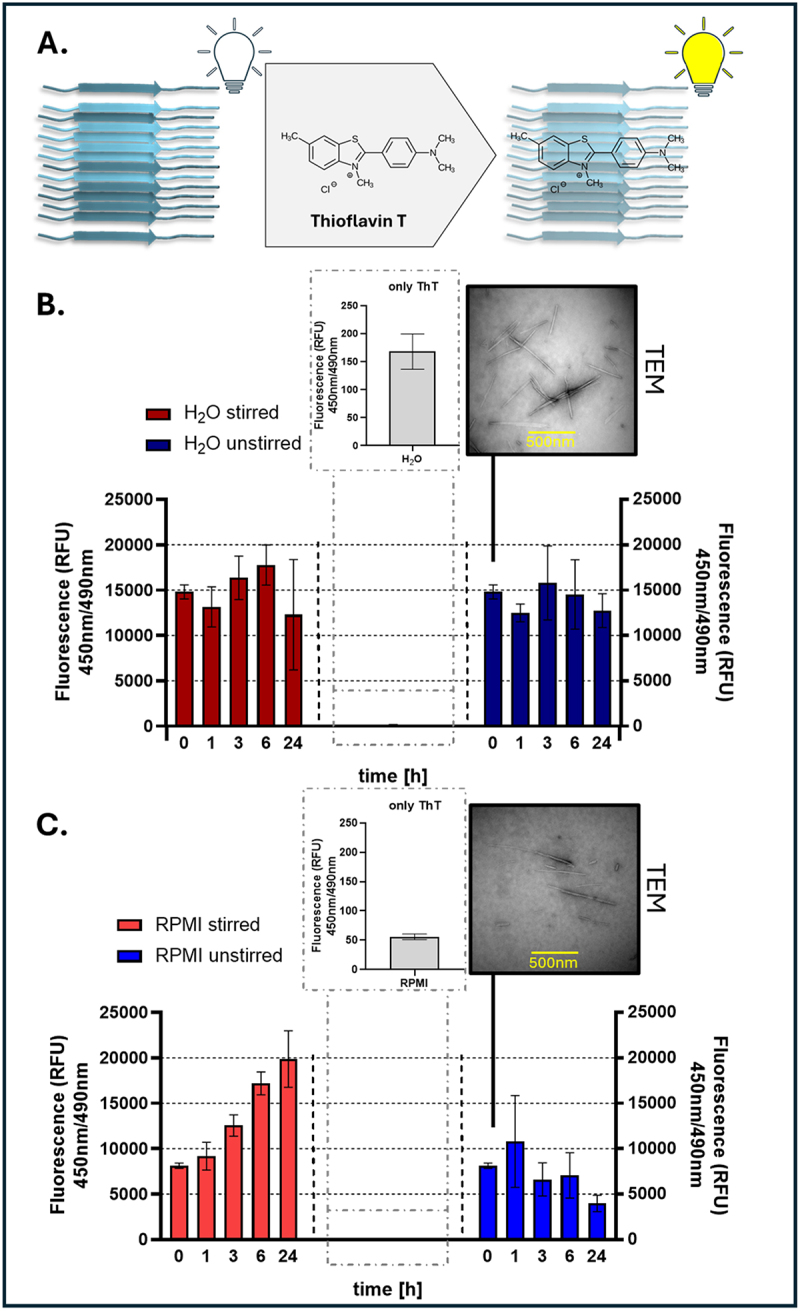
Evaluation of amyloid fibril formation at 4°C agitated and non-agitated conditions by ThT fluorescence measurements and transmission electron microscopy (TEM). (A) principle of ThT fluorescence assay. ThT binds to amyloid fibrils, increasing fluorescence emission (Excitation/emission: 450 nm/490 nm). (B) kinetic analysis of amyloid fibril formation in water under agitated (red bars) and non-agitated (blue bars) conditions. Fluorescence remains at a nearly constant level (slight decrease) over 24 hours when Als5pFP is dissolved in water. TEM image (right) confirms fibrillar structures and the control bar graph (top left) shows minimal signal from ThT alone. (C) amyloid fibril formation in RPMI medium under agitated (red bars) and non-agitated (blue bars) conditions. Agitation enhances fibril formation under agitated conditions, as seen in increased ThT fluorescence. TEM imaging confirmed fibrillar structures. Controls show low background fluorescence from RPMI and ThT alone. Error bars represent standard deviations of experiments conducted in triplicate.

A convenient and easy, but reliable assay for amyloid fibril research is the quantification of fibrillar material using the Thioflavin T (ThT) fluorescence generation assay [77]. ThT measurements were based on the binding of Thioflavin T to the amyloid β-sheet surface, resulting in enhanced [78] fluorescence (Figure 2(A)). To evaluate the fibril-forming capacity of Als5pFP, ThT fluorescence assays were conducted under agitated and non-agitated conditions over 24-hours at 4°C. The ThT fluorescence remained relatively stable in water under agitated and non-agitated conditions, indicating limited but stable fibril formation. This observation was supported by transmission electron microscopy (TEM), which revealed the presence of fibrillar structures directly after dissolving Als5pFP in water (Figure 2(B)). In contrast, incubation in the RPMI medium (clinical standard) resulted in markedly different outcomes. Stirred samples showed a significant increase in ThT fluorescence over time, suggesting enhanced amyloid formation, whereas fluorescence levels in unstirred samples remained comparatively low or even decreased over 24 h, suggesting fibril remodeling or degradation over time. Additionally, TEM analysis confirmed the presence of fibrillar aggregates directly after dissolving Als5pFP in RPMI; however, in contrast to water, more free particles can be assumed to be derived from the complexity of the medium (Figure 2(C)). Control measurements containing ThT alone or ThT in RPMI showed minimal fluorescence, confirming the assay specificity.

**Figure 3.**
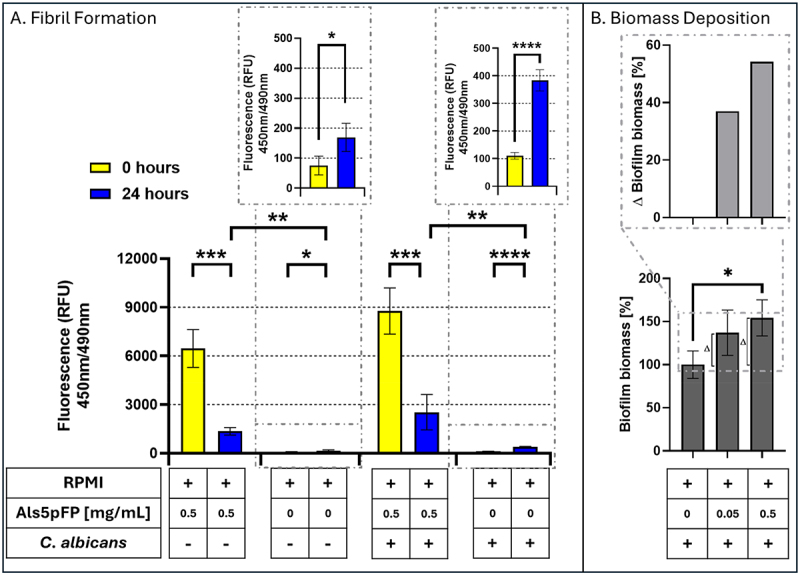
Evaluation of amyloid fibril formation (Als5pFP) in RPMI at 37°C non-agitated condition without and in the presence of *C. albicans* by ThT fluorescence measurements and biomass deposition while exposed to Als5pFP. (A) ThT fluorescence (450 nm/490 nm) was measured at 0 hours (yellow bars) and after 24 hours (blue bars) of incubation with Als5pFP at 37°C and non-agitated conditions (similar to those in biofilm assays). The presence of RPMI medium, Als5pFP and *C. albicans* cells is denoted below the graph. ThT fluorescence significantly decreases over time in conditions containing Als5pFP and C. albicans, indicating amyloid disruption or remodeling. Insets show fluorescence values for Als5pFP negative controls. This experiment was conducted in four replicates. (B) biofilm mass deposition measurement of *C. albicans* exposed to 0 mg/mL, 0.05 mg/mL and 0.5 mg/mL Als5pFP over a period of 24 hours using the crystal violet assay. Top panel: Δ biofilm mass (0.5 mg/mL − 0 mg/mL; 0.05 mg/mL − 0 mg/mL Als5pFP) quantifies the differences in biomass deposition. Error bars represent standard deviations of experiments conducted in triplicate. Statistical analysis was performed using the unpaired Student’s t-test: *p < 0.05, **p < 0.01, ***p < 0.001, ****p < 0.0001.

To mimic the physiological conditions for biofilm formation, the amyloid-forming capacity of Als5pFP was assessed at 37°C under non-agitated conditions in RPMI medium. As shown in Figure 3(A), Als5pFP retained its ability to form amyloid fibrils at 37°C, as indicated by high ThT fluorescence at 0 h. After 24 h, ThT fluorescence decreased in samples containing only Als5pFP, displaying similar effects to those at 4°C and unstirred conditions, as used previously (see Figure 2). When *C. albicans* cells were present, high ThT fluorescence was observed immediately after dissolving the peptide (0 h), indicating preexisting fibrils. However, a significant decrease in fluorescence after 24 h was observed independently of cells being present or absent, suggesting a structural decomposition of the preformed fibrils and thus reduction of ThT stainable material, not only in the presence of cellular metabolism. Despite this reduction, the ThT signal in samples containing only cells without peptide showed a minor increase in fluorescence after 24 h, likely reflecting low but considerable endogenous amyloid production by *C. albicans* under these conditions. Increased biomass deposition as a measure of biofilm formation upon the addition of Als5pFP to *C. albicans* cultures has been observed in a previous study [65]. Using growth conditions that met the clinical standard protocol of the “Clinical & Laboratory Standards Institute” (CLSI, Berwyn, Pennsylvania, USA), the addition of the peptide also resulted in increased and comparable biomass deposition. To determine whether Als5pFP influenced biofilm development, biomass deposition was quantified after 24 h in the presence of increasing concentrations of Als5pFP. As shown in Figure 3(B), treatment with 0.5 mg/mL Als5pFP resulted in a significant (~50%) increase in biofilm mass compared with untreated controls. A modest increase was observed at 0.05 mg/mL. These results indicate that Als5pFP-derived fibrils remain bioactive under the biofilm assay conditions and enhance biofilm accumulation by *C. albicans*.

**Figure 4.**
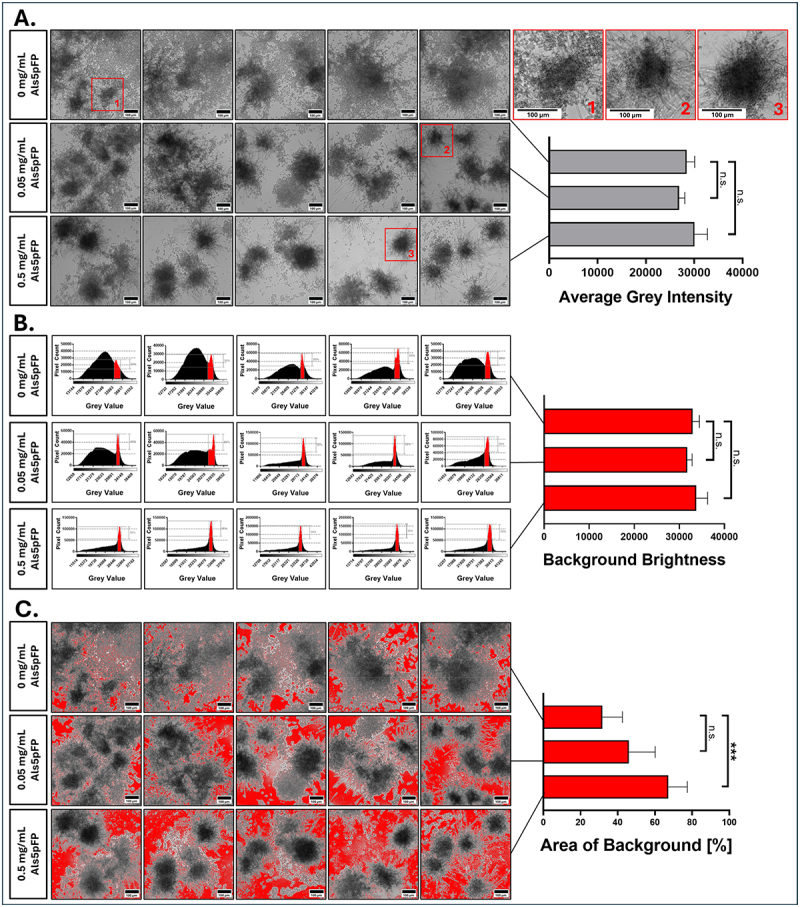
Quantitative image analysis of *C. albicans* biofilm disruption in response to Als5pFP addition. (A) grayscale images of biofilms treated with 0, 0.05 mg/mL or 0.5 mg/mL Als5pFP. Scale bars = 100 µm. The bar graph shows average grey intensity across the five images for each concentration of Als5pFP, indicating overall image brightness. Inlays 1, 2 and 3 show zoomed-in details marked in the images by red squares. (B) histogram analysis focused on the image background areas without visible *C. albicans* cells. The red-marked peak represents this background signal, where only pixel values within the full width at half maximum (FWHM) of this peak were used to generate the adjacent diagram. The diagram shows the average background brightness across five images of each Als5p concentration. The corresponding bar graph quantifies the average background grey intensity. (C) thresholded images showing identified background areas in red (all pixels within the FWHM). A diagram of the quantification (right) of the percentage of image area classified as background of the whole image reveals significantly increased background area with 0.5 mg/mL Als5p, suggesting shape-edging of microcolony formation. Statistical analysis was performed using the unpaired Student’s t-test: ***p < 0.001, n.S. = not significant.

Based on previous data confirming the presence of amyloid fibrils after 24 h under biofilm-relevant conditions (37°C, not agitated, and in RPMI medium), we next investigated the structural consequences of these fibrils on *C. albicans* biofilm architecture using quantitative image analysis. The biofilms were allowed to form over 24 h in the presence of 0, 0.05, or 0.5 mg/mL Als5pFP. As shown in Figure 4A, the average image brightness (mean gray value) across all images remained unchanged between the three conditions, verifying the intended comparable signal levels and no major differences in image exposure and identical physical illumination. Similarly, the analysis of background gray intensity (Figure 4(B)), based on the full width at half maximum (FWHM) of the background peak in the histogram, showed no significant difference in the mean background brightness between the three concentrations. However, when quantifying the proportion of the image area already classified as background, a significant increase in background area was detected in the 0.5 mg/mL Als5pFP condition compared to the control (0 mg/mL) (Figure 4(C)). This indicated a more defined and less spatially organized biofilm architecture, likely suggesting a considerable shape-edging of microcolony borders as a consequence of the presence of fibril-forming Als5pFP. The 0.05 mg/mL condition showed no significant difference compared with the control.

**Figure 5.**
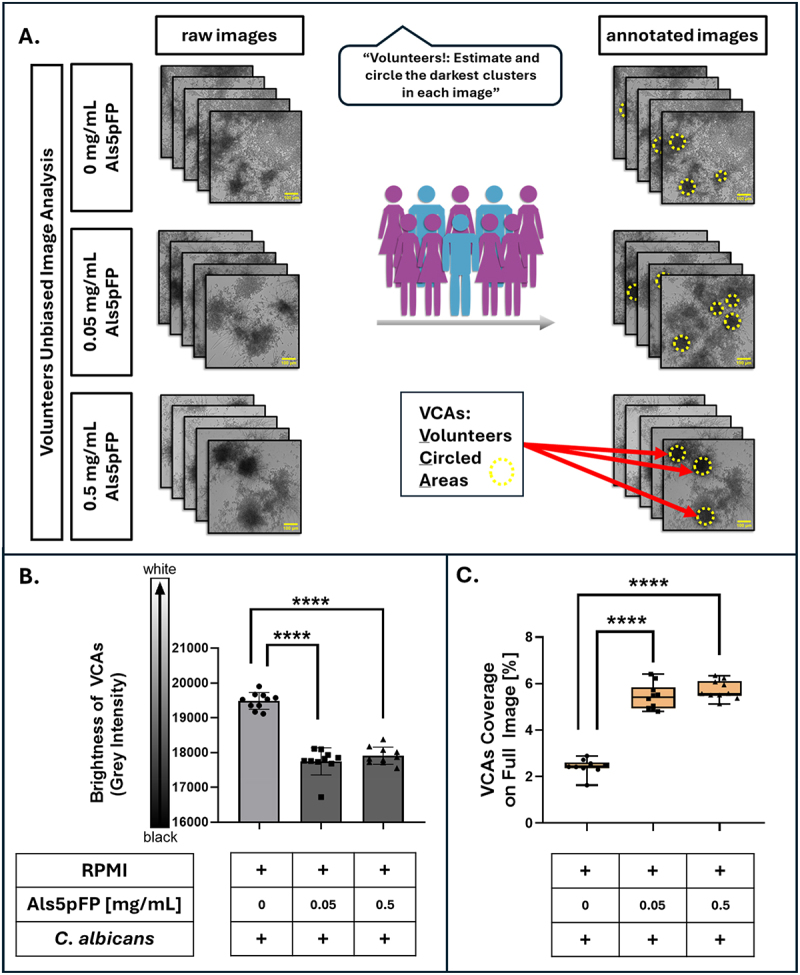
Volunteers unbiased image analysis reveals Als5pFP dependent microcolony shape-edging. (A) 10 volunteers were asked to identify and circle the darkest cluster(s) in each image of biofilms treated with 0, 0.05 or 0.5 mg/mL Als5p. Selected regions (yellow dotted circles) reflect the visual consensus of biofilm density and are labeled as volunteers circled areas (VCAs) (B) the average grey intensity of VCAs decreases significantly with increasing Als5pFP concentration, indicating the creation of dense biofilm clusters (from white low-density biofilm to dark high-density biofilm). (C) the area of the VCAs was summed for each image and the percentage of the total circled area relative to the entire image was calculated. This analysis confirms an increase in the darkest regions of images with higher Als5pFP treatment error bars represent standard deviations of experiments conducted in triplicate. Statistical analysis was performed using the unpaired Student’s t-test: ****p < 0.0001.

To test the hypothesis that Als5pFP treatment induces alterations in biofilm architecture by forcing cells to form more compact (microcolony) structures, we combined the easy image analysis procedure with a “Citizen Science” approach. Structural changes were quantified thereby in an unbiased manner, by analyzing microscopic images of *C. albicans* biofilms treated with 0, 0.05 or 0.5 mg/mL Als5pFP by volunteers. The idea behind this concept was to involve non-experts in the analysis to ensure an unbiased view of the individual sets of images to be analyzed. As illustrated in Figure 5(A), ten independent volunteers with scientific backgrounds but without dedicated knowledge of the specific hypothesis behind the experimental approach were asked to manually annotate the darkest visible cluster (s) in each image using a standardized ImageJ macro. These manually selected regions were termed Volunteers Circled Areas (VCAs) and represented the volunteers’ perception of the densest microcolonies based on their darkness. Quantification of brightness within the VCAs (Figure 5(B)) revealed a significant decrease when Als5pFP was added. Because lower gray values indicate darker regions, this result demonstrates that the circled clusters upon Als5pFP addition were visually and quantitatively darker. This suggests the emergence of denser, more compact biofilm regions with increased fibril numbers. Each symbol in the plot (circle, triangle, or square) represents a single volunteer’s selection, confirming that this trend was consistently perceived by all participants. Furthermore, analysis of the total area of the VCAs relative to the entire image (Figure 5(C)) showed a significant increase at 0.05 mg/mL and 0.5 mg/mL Als5pFP compared to the control. At 0 mg/mL, the low number or absence of clear dark clusters likely led volunteers to either omit selections or include relatively lighter regions (Figure 5(B)), resulting in smaller and more variable total VCAs. In contrast, a higher Als5pFP concentration led to more consistent and larger annotated regions, further supporting the idea of microcolony shape-edging. These findings indicate that Als5pFP treatment promotes the formation of darker and more spatially defined microcolonies, a conclusion independently derived from annotations based on estimations of microcolony borders by multiple unbiased observers.

## Discussion

The ability to form elaborate architectures in mature biofilms represents a valuable option for pathogenic cells, including *C. albicans* to hide from the immune system and establish a growing resistance against chemical compounds such as antifungal drugs, leading to considerable health threats and therapeutic challenges (nicely reviewed in Amann et al. [79]). The mode of action is realized simply by creating areas of high cell density covered with extracellular materials that act as molecular sieves to limit the diffusion of toxic material into direct contact with cells. In this context special physiological importance lies in microcolonies serving as “seeds” for increasing resistance and thus pathogenic potential. Microcolony formation occurs in the early phases of biofilm formation and is characterized by an already occurring resistance against antifungal agents, such as fluconazole, which is then surmounted to full resistance (up to 1000-fold) in mature biofilms [80]. Consequently, microcolonies as the physiological structural and morphological foundation for the successful establishment of elaborate mature biofilms that provide *C. albicans* with its full pathogenic potential and virulence capabilities deserve the highest attention not only for understanding further biofilm development, but also for the development of potential new strategies in the fight against *C. albicans* infections. We appreciate the fact that, in principle, every factor influencing microcolony formation, their outgrowth into mature architectures in biofilms or, in contrast, the dissolution of biofilms or sub-mature biofilm structures represent important potential “adjusting screws” in the fight against biofilms. In this light the finding of Lipke and coworkers that the Als5p derived peptide Als5pFP can increase biomass deposition into biofilms in cultures of *C. albicans* already qualifies this peptide as an important molecular player in biofilm formation [65]. Our findings, based on a novel variant of an easy image analysis applied to microscopic photographs of microcolonies observed from growing cultures of *C. albicans* growing in the presence or absence of Als5pFP, not only substantiate evidence for Als5pFP influence on biofilm formation *per se* but also represent an amendment to understand its molecular role by demonstrating for the first time that microcolony development is influenced by concentrating more cells into these structures accompanied by a significant reduction of previously not yet organized single cells in the interspace between microcolonies. The apparent circular or rounded morphology of *C. albicans* biofilms observed upon addition of Als5pFP likely suggests an isotropic reorganization of cell-cell adhesion forces induced by the peptide´s amyloid fibrils. Als5pFP promotes the formation of fibrillar networks that may interconnect neighboring cells evenly in all spatial directions. This isotropic strengthening of intercellular adhesion may minimize structural irregularities and causes the aggregation of cells into compact, radially, hemispherical symmetric microcolonies [81]. Such symmetric growth pattern is expected when adhesion and matrix cohesion act equally in all directions. It has been shown when there is a high nutrients level the growth pattern is round and compact whereas for decreasing nutrient levels the biofilms form more like fractal growth patterns [82]. Therefore, the observed “circular” morphology is not due to any inherent circular geometry of the fibrils themselves, but rather to a uniform distribution of amyloid-mediated adhesive forces within the developing microcolony. Amyloid-like structures like the so-called “curli fibrils” produced by bacterial pathogens including *Salmonella Typhimurium* and *Escherichia coli*, have been described [83–85] as significantly influential for the formation of respective biofilms. Interestingly, the concept of a physiological “concentration” of human cells and infective virus particles, in this case the HI-Virus, has been described for amyloid fibrils [86], which form molecular meshes, forcing the virus into close local contact with the target cells to be infected, thereby drastically enhancing its infectivity. In contrast to these directly measurable positive effects of amyloids on virus infectivity, it appears challenging and of high interest to address the direct effects of Als5pFP on the virulence of *C. albicans*, as well as to determine the potential influence on the development of antifungal resistance in the microcolony seeds shape-edged by g Als5pFP. Moreover, based on the knowledge of curli fibrils, it is of interest to clarify their potential direct effects on inflammation. Research has shown that curli fibrils not only contribute to biofilm architecture but can also act as danger signals by activating dendritic cells and triggering the production of pro-inflammatory cytokines such as IL-6, TNF-α, and IL-12. So-called curli-DNA composites, complexes of amyloid curli fibers, and bacterial extracellular DNA that naturally occur in biofilms have a particularly immunostimulatory effect. These complexes promote the maturation and activation of antigen-presenting cells via type I interferon signaling and can exacerbate autoimmune processes, such as those observed in systemic lupus erythematosus (SLE) [87].

## Conclusion

In summary, we have shown that the Als5pFP peptide can form amyloid-like fibrils in the presence of growing *C. albicans* cells, leading to a concentration of cells in morphologically shape-edged microcolonies, thereby addressing the potential role of the peptide during the events required for the development of architectural mature biofilms of this highly relevant human pathogen. We hope to have opened a new avenue toward the development of Als5p directed drugs for the inhibition of biofilm formation and the development of biosensors that directly allow the kinetic measurement of amyloid formation and dissolution by future drug molecules.

### Informed consent statement

Not applicable.

## Supplementary Material

editable figures.pptx

## Data Availability

Data are available on https://data.mendeley.com/datasets/xwdrm3hj8x/1 [82].
