# Supplementary material for: Amyloid fibrils of the Als5p-derived peptide NH2-SNGIVIVATTRTV-COOH influence the biofilm formation of Candida albicans by shape-edging microcolony morphology
Source: Virulence. 2025 Dec 10;16(1):2597576. doi: 10.1080/21505594.2025.2597576 (PMC12698048; doi:10.1080/21505594.2025.2597576)
Supplement: editable figures.pptx [file KVIR_A_2597576_SM7029.pptx]

## Slide 1
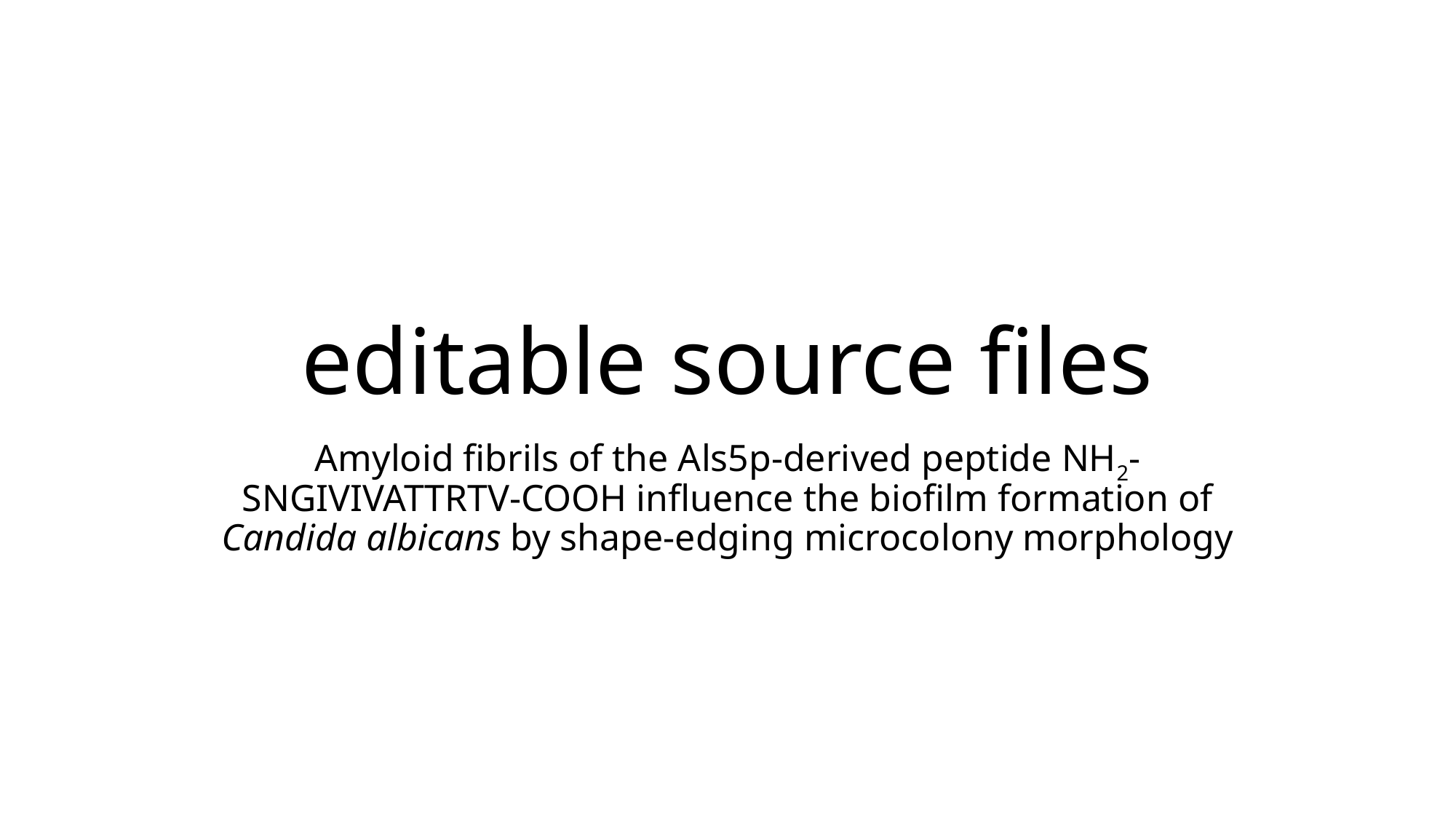

# editable source files
Amyloid fibrils of the Als5p-derived peptide NH2-SNGIVIVATTRTV-COOH influence the biofilm formation of Candida albicans by shape-edging microcolony morphology

## Slide 2
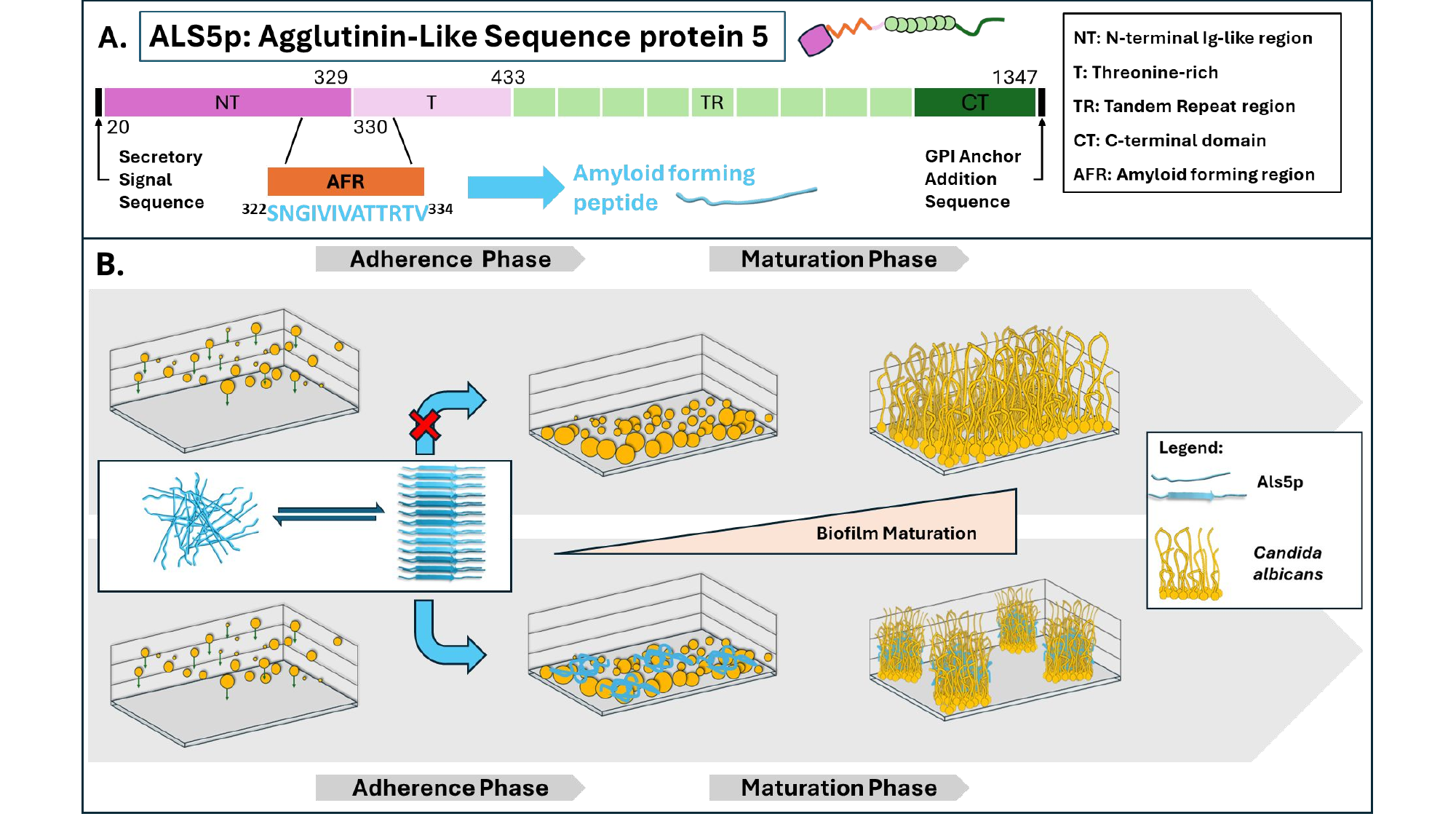

B.

## Slide 3
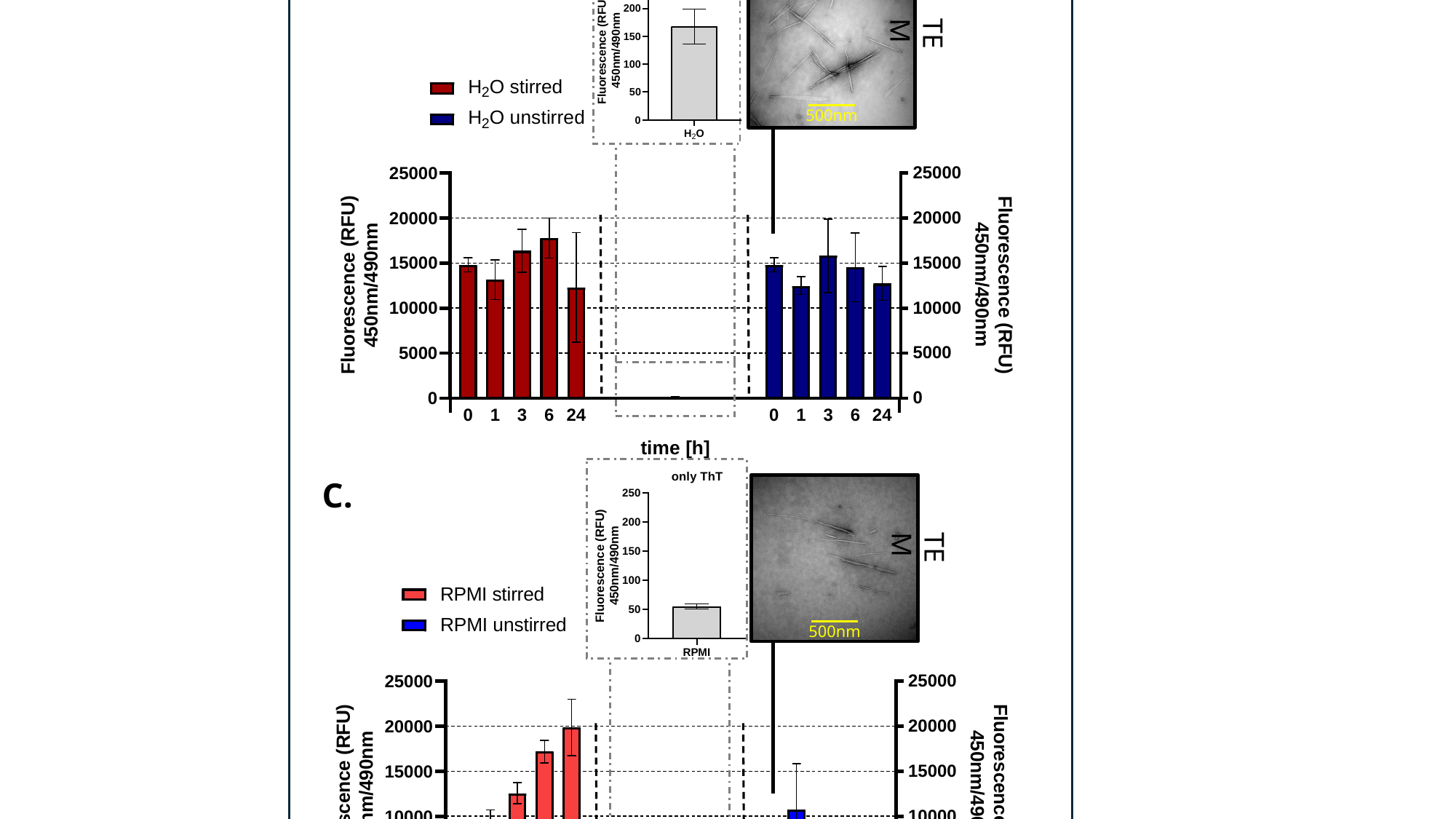

A.
B.
TEM
500nm
C.
TEM
500nm

## Slide 4
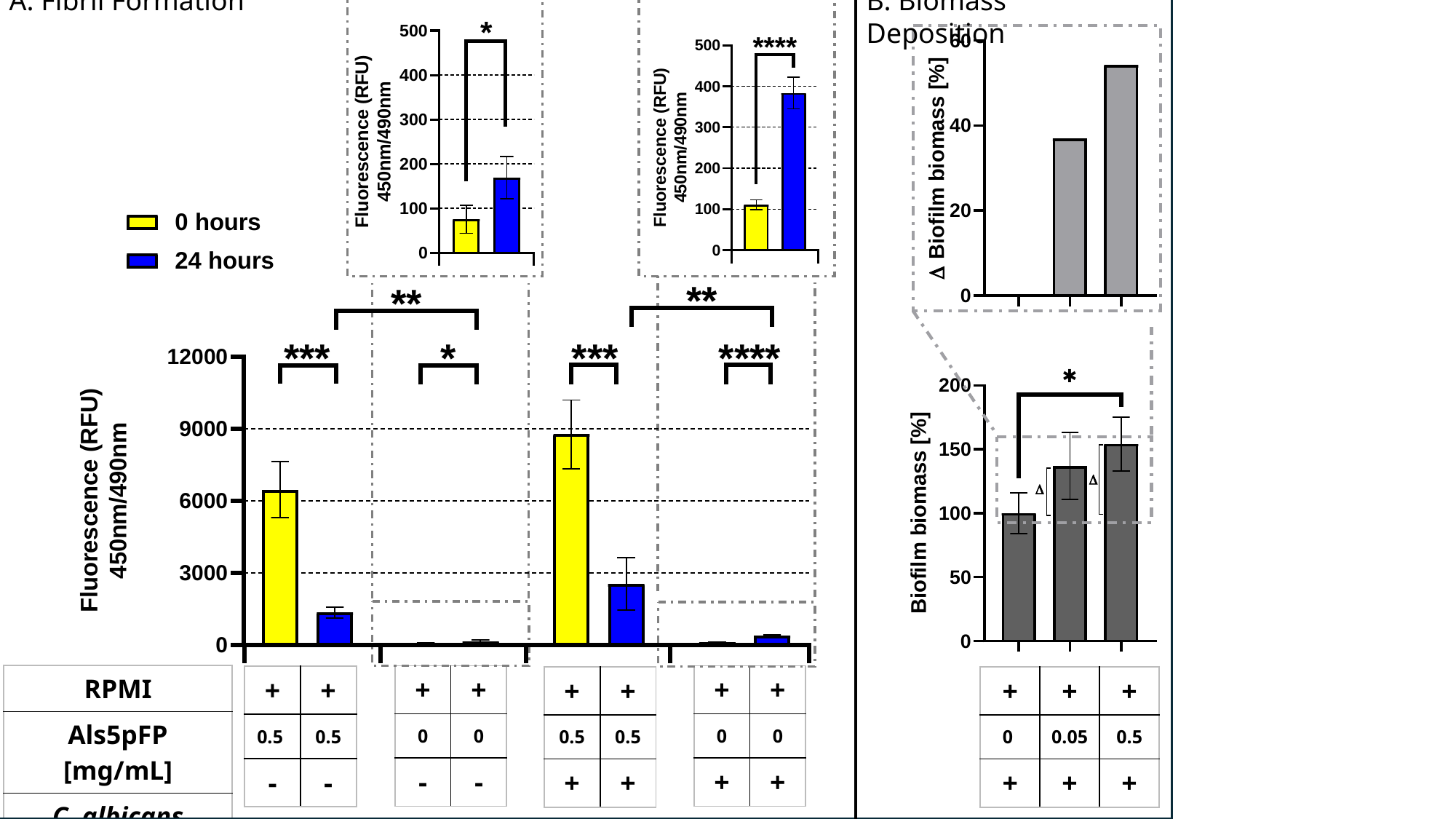

A. Fibril Formation
B. Biomass Deposition
| RPMI |
| --- |
| Als5pFP [mg/mL] |
| C. albicans |
| + | + |
| --- | --- |
| 0 | 0 |
| - | - |
| + | + |
| --- | --- |
| 0 | 0 |
| + | + |
| + | + |
| --- | --- |
| 0.5 | 0.5 |
| - | - |
| + | + |
| --- | --- |
| 0.5 | 0.5 |
| + | + |
| + | + | + |
| --- | --- | --- |
| 0 | 0.05 | 0.5 |
| + | + | + |

## Slide 5
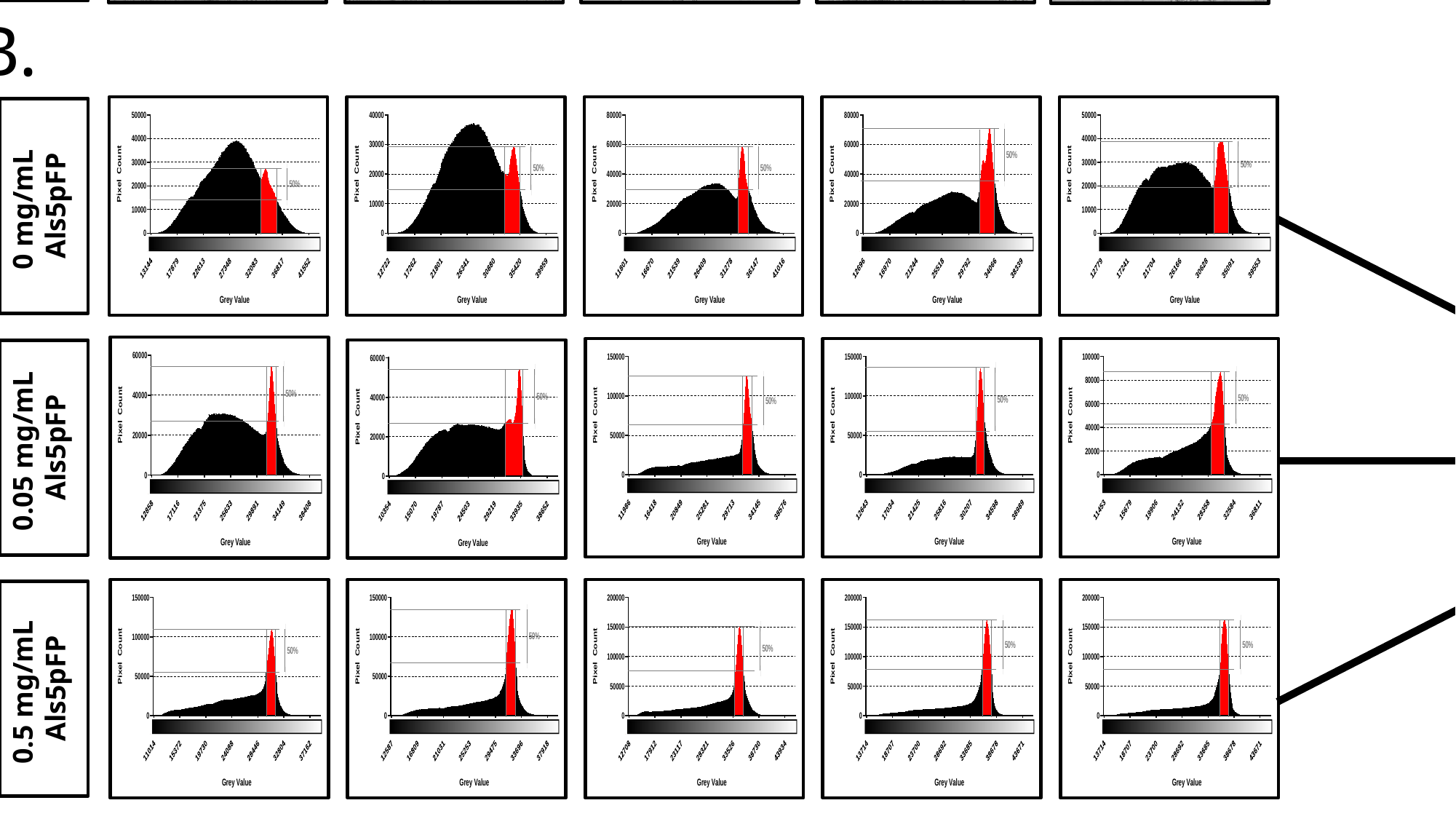

A.
0 mg/mL
Als5pFP
0.05 mg/mL
Als5pFP
0.5 mg/mL
Als5pFP
B.
0 mg/mL
Als5pFP
0.05 mg/mL
Als5pFP
0.5 mg/mL
Als5pFP
C.
0 mg/mL
Als5pFP
0.05 mg/mL
Als5pFP
0.5 mg/mL
Als5pFP

## Slide 6
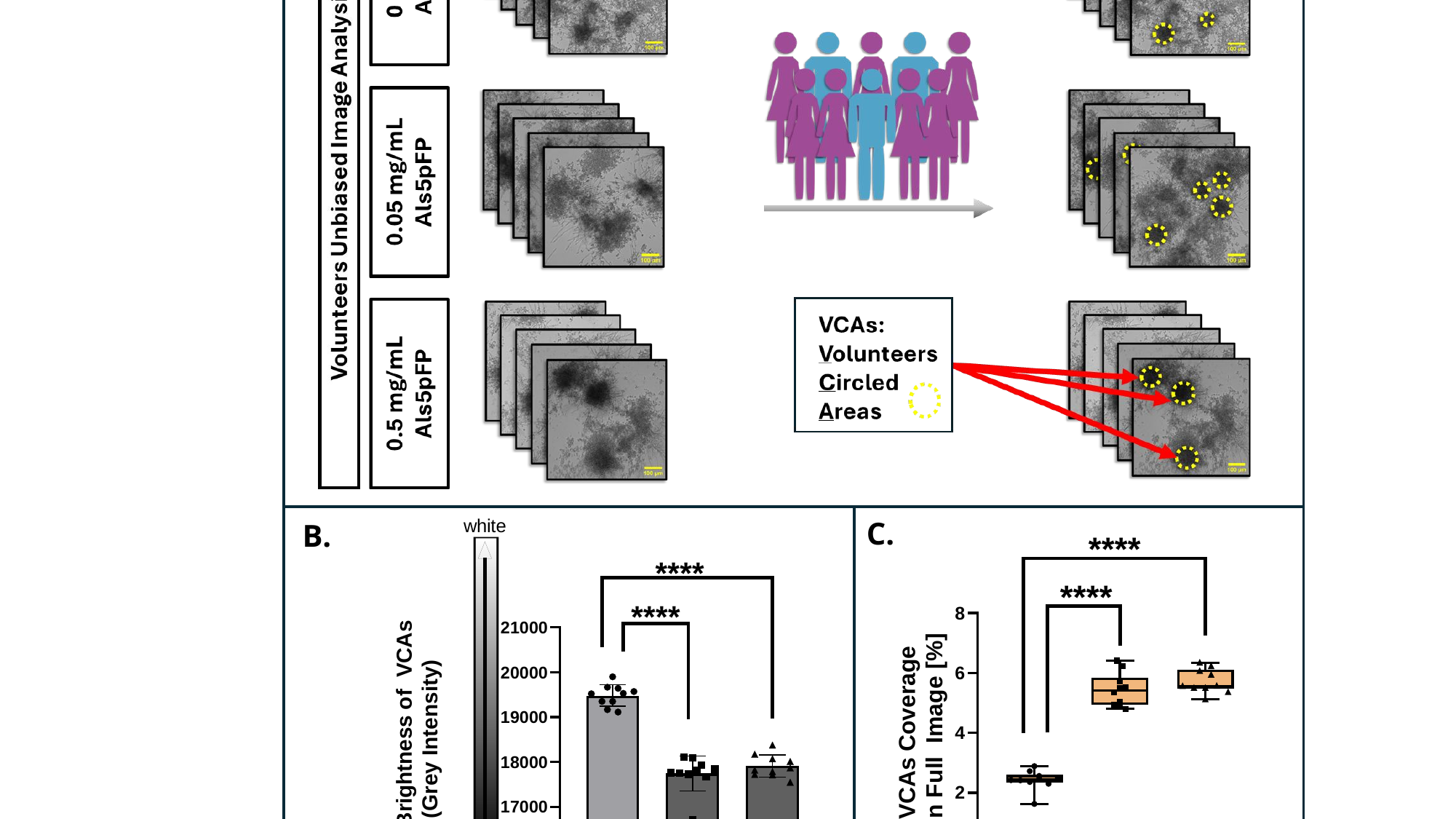

A.
C.
B.
| + | + | + |
| --- | --- | --- |
| 0 | 0.05 | 0.5 |
| + | + | + |
| RPMI |
| --- |
| Als5pFP [mg/mL] |
| C. albicans |
| + | + | + |
| --- | --- | --- |
| 0 | 0.05 | 0.5 |
| + | + | + |
